# Population characteristics, glucocorticoid dosage, and risk factors for osteonecrosis of the femoral head in systemic lupus erythematosus: a Systematic Review and meta-analysis

**DOI:** 10.3389/fimmu.2026.1755818

**Published:** 2026-02-11

**Authors:** Jiawen Zhang, Jindong Zhang, Yawei Dong, Nongyi Li, Junjie Li, Yishu Wen, Jia Yan, Biao Tan, Yan Yan

**Affiliations:** 1 The Third Affiliated Hospital of Beijing University of Chinese Medicine, Beijing, China; 2 Chongqing University of Chinese Medicine, Chongqing, China; 3 Ningxia Zhang’s Orthopedic Hospital, Yinchuan, Ningxia, China; 4 Wangjing Hospital of China Academy of Chinese Medical Sciences, Beijing, China; 5 Shanxi Provincial Bethune Hospital, Taiyuan, Shanxi, China; 6 The First Affiliated Hospital of Chongqing University of Chinese Medicine, Chongqing, China

**Keywords:** glucocorticoid dosage, osteonecrosis of the femoral head, population characteristics, risk factors, systemic lupus erythematosus

## Abstract

**Background:**

Osteonecrosis of the femoral head (ONFH) is a severe complication of systemic lupus erythematosus (SLE). However, the demographic characteristics, glucocorticoids (GCs) risks, and other contributing factors remain debated.

**Objective:**

To elucidate the population characteristics, GCs-related risks, and other risk factors for ONFH in patients with SLE through a systematic review and meta-analysis, thereby enhancing the clinical identification of high-risk populations and optimizing GCs therapy strategies in SLE.

**Methods:**

We searched seven databases, from their inception until July 2025 for relevant cohort and case-control studies. The quality of included studies was assessed using the Newcastle-Ottawa Scale. Meta-analysis was performed using RevMan 5.3.

**Results:**

Thirty-five studies involving 11,356 participants were included. Regarding population characteristics, patients with SLE who developed ONFH had a significantly younger age at diagnosis (SMD = -0.19, *P* < 0.00001) and higher SLEDAI scores (SMD = 0.21, *P* = 0.002). Among metabolic and immune indicators, elevated triglycerides (SMD = 0.21, *P* = 0.02), decreased high-density lipoprotein cholesterol (SMD = -0.22, *P* = 0.03), and positive antiphospholipid antibodies (OR = 2.00, *P* = 0.04) were associated with ONFH occurrence. Regarding GC therapy, pulse steroid therapy (OR = 2.02, *P* < 0.00001), an initial dose >60 mg/day (OR = 4.19, P < 0.0001), a maximum daily dose >50 mg (SMD = 0.42, *P* = 0.0002), and higher average daily GC intake (SMD = 0.32, *P* = 0.004) significantly increased ONFH risk. In contrast, cumulative GC dose showed no significant association (*P* = 0.14). Furthermore, vasculitis (OR = 3.17, *P* < 0.00001), hypertension (OR = 1.48, *P* = 0.02), Raynaud’s phenomenon (OR = 1.60, *P* = 0.0003), thrombocytopenia (OR = 1.69, *P* = 0.007), and arthritis (OR = 1.88, *P* = 0.006) were identified as independent risk factors.

**Conclusion:**

Patients with SLE at high risk for ONFH exhibit distinct characteristics. Short-term high-dose GC exposure, rather than cumulative dose, constitutes the core medication-related risk. Enhanced imaging screening and comprehensive, multi-factorial prevention strategies are warranted, particularly for patients receiving high initial doses or pulse therapy. Clinical management should focus on optimizing GC regimens in these high-risk individuals to minimize the occurrence of ONFH.

**Systematic review registration:**

https://www.crd.york.ac.uk/PROSPERO/view/CRD420251084371, identifier CRD420251084371.

## Introduction

Systemic lupus erythematosus (SLE) is a chronic, relapsing-remitting inflammatory disease characterized by aberrant autoimmune responses. Its pathogenesis involves genetic susceptibility, environmental factors, sex hormones, and immune dysregulation (1). SLE typically affects multiple organs and systems, including the skin, joints, kidneys, cardiovascular system, and central nervous system (2). With the optimized use of treatments such as glucocorticoids (GCs), immunosuppressants, and biologic agents, the long-term prognosis of SLE patients has significantly improved, with current 10-year survival rates exceeding 90% (3). However, as patient survival increases, chronic disease damage and treatment-related adverse effects have become major concerns impacting quality of life and long-term outcomes. Chronic renal insufficiency, cardiovascular disease, and musculoskeletal complications are increasingly prominent (4).

Among musculoskeletal disorders, osteonecrosis (ON) is one of the most severe and common complications in SLE patients. Epidemiological studies indicate that the incidence of ON in SLE ranges from approximately 1.7% to 52%, far exceeding that in the general population (5). Steroid-induced osteonecrosis of the femoral head (SONFH) is the most prevalent form. The disease often has an insidious onset, frequently involves both hips, and may be asymptomatic in early stages. If missed during the optimal intervention window, the necrotic area typically progresses, eventually leading to femoral head collapse and severe hip dysfunction. Many patients consequently require total hip arthroplasty (THA), imposing a substantial burden on both individual quality of life and the healthcare system.

The pathogenesis of ONFH in patients with SLE is complex, with long-term and high-dose GCs therapy recognized as the predominant risk factor (6). GCs are thought to promote ONFH through multiple pathways, including inducing the differentiation of bone marrow adipocytes, impairing vascular endothelial function, suppressing osteoblast activity, and causing dyslipidemia (7, 8). Furthermore, combination therapy with immunosuppressants (9), the presence of antiphospholipid antibodies (10), and dyslipidemia (11) may also increase the risk of localized microcirculatory impairment and necrosis in the femoral head.

However, despite the established relationship between GCs and ONFH, inconsistencies exist regarding the roles of cumulative dose, daily dosage, pulse therapy regimens, and treatment duration (12, 13). Some studies suggest that high-dose pulse therapy significantly increases risk (14), while others indicate that the cumulative dose during maintenance therapy is more decisive (15). This lack of consistent and sufficient evidence directly hampers evidence-based clinical risk stratification and medication management. On one hand, some patients develop ONFH even with low-to-moderate dose GCs, whereas others do not despite receiving high-dose treatment, implying that underlying high-risk patient profiles are not yet fully identified. On the other hand, the absence of systematically integrated evidence makes it challenging for clinicians to accurately assess an individual’s true risk of developing ONFH when balancing disease control against potential adverse effects. This not only complicates early diagnosis and prevention but also causes some patients to miss the optimal window for intervention, ultimately resulting in a substantial burden for both the individual and society.

Therefore, this study intends to conduct a systematic review and meta-analysis to consolidate existing evidence, aiming to clarify the demographic characteristics and GCs dosing patterns associated with ONFH in SLE, and to explore the relationships between other potential risk factors and ONFH development. The findings are expected to enhance the clinical identification of high-risk populations, optimize GCs application strategies in SLE patients, facilitate the implementation of early screening and interventions, and provide an evidence-based foundation for subsequent mechanistic research and the refinement of treatment protocols.

## Method

This study has been registered in the PROSPERO(CRD420251084371), in accordance with the Preferred Reporting Items for Systematic Reviews and Meta-Analyses (PRISMA) criteria (16).

### Search strategy and study selection

The present study included cohort and case–control studies on ONFH complicating SLE published from inception until July 2025. The databases searched included Pubmed, Embase, Web of Science, Ovid Technologies, Cochrane library database, China National Knowledge Infrastructure(CNKI), Chinese Science and Technology Periodical Database, and Wanfang Database. This search strategy employed Medical Subject Headings terms and relevant keywords to identify studies examining the demographic characteristics, corticosteroid dosage, and associations with potential risk factors in patients with SLE complicated by ONFH. Please refer to **Supplementary Material 1** for the complete search strategy used in every database.

Two authors independently screened all titles and abstracts based on the inclusion and exclusion criteria. Disagreements at this stage were resolved by a third author. Subsequently, the full texts of potentially eligible studies were assessed for final inclusion. Any discrepancies arising throughout the process were resolved through consensus.

### Inclusion and exclusion criteria

#### Inclusion criteria

1. Chinese or English.
2. Case-control studies and cohort studies
3. Study subjects are patients with a confirmed diagnosis of SLE who are receiving GCs therapy, with the study protocol detailing the specifics of GCs treatment.
4. Follow-up reports should include content related to ONFH confirmed by imaging.
5. Reports should include the type of GCs, route of administration, daily dose, maximum dose, cumulative dose (converted to prednisone equivalents if applicable), duration of GCs therapy, and outcomes related to the occurrence of osteonecrosis.
6. Report potential risk factors for osteonecrosis.
7. Sufficient published data are available to estimate the hazard ratio (HR)/odds ratio (OR) or weighted mean difference (WMD) and 95% confidence interval (CI).

#### Exclusion criteria

1. Bone necrosis associated with tumors or infections.
2. Animal studies, biomarker studies, conference abstracts, non-original studies (e.g., editorials, literature reviews, commentaries, protocols, and guidelines).
3. Data with incomplete or unrecoverable outcome measures and duplicate data.

### Data extraction

All studies included in this analysis incorporated the following variables: first author, publication year, location, study type, number of participants, age, gender, and diagnostic method for ONFH. Additionally, factors mentioned in the papers—such as the maximum dose, average dose, cumulative dose, duration of GCs therapy, and associated risk factors in SLE patients developing ONFH following GCs treatment—were extracted as study outcomes.

The mean and standard deviation for each factor, along with the odds ratio (OR) associated with ONFH occurrence, were extracted from each paper. Based on the OR values extracted from each paper, the logarithmic odds ratio (logOR) and standard error (SE) were calculated.

### Quality assessment

The Newcastle-Ottawa Scale (NOS) (17) was used to assess the quality of the included case-control and cohort studies. The scale comprises three dimensions: Selection, Comparability, and Exposure. A study could receive a maximum of one star for each item within the Selection and Exposure categories. Regarding Comparability, a maximum of two stars could be awarded. Each star corresponds to one point. The average scores for the included case-control studies and cohort studies were calculated separately.

### Statistical analysis

Statistical analyses were performed using RevMan software (version 5.3; The Cochrane Collaboration, London, UK). We extracted mean values and standard deviations for each variable from the original studies to assess the demographic characteristics and corticosteroid dosage in the population with ONFH complicating SLE. Concurrently, adjusted ORs (from multivariate analysis models) and their 95% confidence intervals (CIs) were extracted. Where studies provided both unadjusted ORs and ORs adjusted for potential confounders, the adjusted ORs were used to assess the association between different variables and the risk of ONFH. The *I*² statistic was calculated to assess the proportion of total variation attributable to inter-study heterogeneity. An I² value exceeding 50% indicates moderate to high heterogeneity. Owing to the anticipated heterogeneity in study designs and variations in corticosteroid standardization, a random-effects model was employed for all analyses, regardless of the *I*² value.

For any variable presenting with large heterogeneity, a sensitive analysis excluding outlier studies was conducted to investigate the potential sources of heterogeneity. We attempt to assess the possibility of publication bias by constructing a funnel plot of each trial’s effect size against the standard error.

## Results

### Literature search and screening

**Figure 1:**
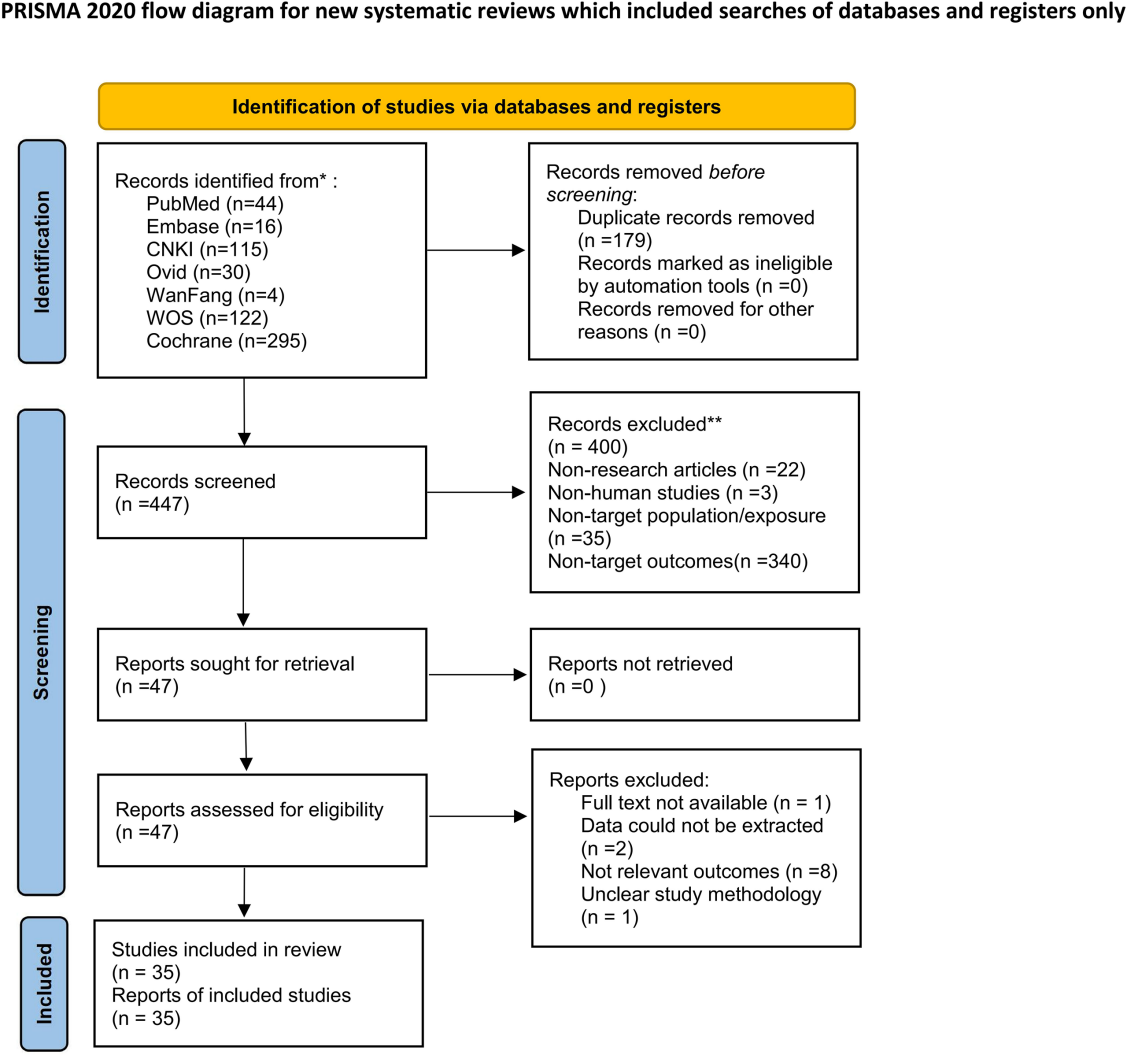
Flowchart of study selection.

From the electronic search conducted, 526 papers were identified from the seven databases, in total, 35 papers (10, 18–51) (11,356 participants) were included in this review (**Figure 1**).

### Assessment of quality

27 case–control studies and 8 cohort studies were included in all of the 35 papers included in this review. A lack of Selection of Controls and incomplete information about the Non-Response rate were the most common shortcomings in these studies. All included case-control studies scored 5.2, while all included cohort studies scored 6. **Supplementary Material 2: Table S1-S2** show a detailed assessment using the NOS Assessment Scale.

### Characteristics of the eligible studies

The 35 studies included in this review were performed in 12 different countries. 27 case–control studies and 8 cohort studies were included in this review, which contains 11,356 participants. Variables include gender, age at onset of SLE, disease activity, specific organ involvement, and detailed GCs exposure metrics. The main characteristics of the trials are summarized in **Table 1**.

**Table 1:** Characteristics of the included studies.

Reference	Region/year	Study design	Sample size(cases)	Number of ONFH(n)	GC	GC dose conversion	Diagnosis of ONFH	Risk factors
Al Saleh J (18)	United Arab Emirates/2010	Case control	126	21	prednisone	/	/	Gender
Calvo-Alén J (19)	USA/2006	Case control	92	33	glucocorticoids	/	X-ray or MRI	BMI、Gender、TC、TG、HDL-C、LDL-C、Duration of SLE、Daily GC Consumption、Maximum Daily Dose of GC>50mg
Davidson J E (20)	USA/2018	Case control	114	38	glucocorticoids	prednisone	/	Gender、Duration of SLE、Cumulative dose of GC、SLEDA score
Faezi S T (21)	Iran/2015	Case control	665	105	glucocorticoids	/	MRI	Age at SLE Diagnosis、Gender、Anti-phospholipid antibodies、Cumulative dose of GC、SLEDA score
Gontero RP (22)	Argentina/2015	Case control	158	15	glucocorticoids	prednisone	X-ray, CT or MRI	Raynaud's Phenomenon、Vasculitis、Thrombocytopenia、Hypertension
HAMIJOYO L (23)	Filipino/2008	Case control	136	43	prednisone	/	X-ray or MRI	Raynaud's Phenomenon、Vasculitis
Hamza SM (24)	Egypt/2019	Case control	60	30	glucocorticoids	/	MRI	Gender、TC、TG、Duration of SLE、SLEDAl score
Kunyakham W (25)	Thailand/2012	Case control	736	65	glucocorticoids	prednisone	X-ray or MRI	Age at SLE Diagnosis、Gender、Arthritis、Vasculitis、Hyperension、Duration of SLE
Lee J (26)	Korea/2014	Case control	128	64	glucocorticoids	prednisone	X-ray or MRI	Age at SLE Diagnosis、Gender、Duration of SLE、Daily GC Consumption、Raynaud's Phenomenon、Vasculitis、Thrombocytopenia、GC treatment duration、Glucocorticoid pulse therapy、Cumulative dose of GC
Li JF (27)	China/2014	Case control	98	40	glucocorticoids	prednisone	X-ray, CT or MRI	Age at SLE Diagnosis、Gender、Duration of SLE、Vasculitis、Raynaud's Phenomenon、Thrombocytopenia、Daily GC Consumption、GC treatment duration、Glucocorticoid pulse therapy、Cumulative dose of GC
Lin J (28)	China/2014	Case control	52	26	glucocorticoids	prednisone	/	Gender、TG、TC、Raynaud's Phenomenon、Glucocorticoid pulse therapy、GC Starting Dose、SLEDAl score
Li SY (29)	China/2008	Case control	64	32	prednisone	/	/	Duration of SLE、TC、TG、HDL-C、LDL-C、Anti-phospholipid antibodies、Vasculitis、Maximum Daily Dose of GC >50mg、Glucocorticoid pulse therapy、Cumulative dose of GC、GC Starting Dose、SLEDAl score、
Liu ZY (30)	China/2011	Case control	80	40	prednisone	/	/	Gender、TG、TC、Anti-phospholipid antibodies、LDL-C、Vasculitis、Raynaud's Phenomenon、Glucocorticoid pulse therapy、GC Starting Dose
Mok CC (31)	China/1998	Case control	181	38	glucocorticoids	prednisone	X-ray or MRI	Gender、Duration of SLE、Thrombocytopenia、Hypertension、Raynaud's Phenomenon、GC treatment duration、Glucocorticoid pulse therapy、Cumulative dose of GC
Qi YQ (32)	China/2010	Case control	376	27	glucocorticoids	/	MRI	Gender、Duration of SLE、TC、TG、LDL-C、Raynaud's Phenomenon、Vasculitis、Hypertension
Sayarlioglu M (33)	Turkey/2012	Case control	203	49	glucocorticoids	prednisone	X-ray, radioisotope bone scan or MRI	Age at SLE Diagnosis、Gender、Anti-phospholipid antibodies、Vasculitis、Raynaud's Phenomenon、Thrombocytopenia、Hypertension、Daily GC Consumption、GC treatment duration、Cumulative dose of GC
Shen LX (34)	China/2005	Case control	262	28	glucocorticoids	/	X-ray, CT or MRI	Gender、Duration of SLE、Vasculitis、Raynaud's Phenomenon、Anti-phospholipid antibodies、Daily GC consumption、GC treatment duration、Glucocorticoid pulse therapy
Shen MN (35)	China/2012	Case control	40	10	glucocorticoids	prednisone	X-ray, CT or MRI	Duration of SLE、TC、TG、HDL-C、LDL-C、Glucocorticoid pulse therapy、Cumulative dose of GC、SLEDA score
Shi YJ (36)	China/2013	Case control	84	42	prednisone	/	/	Gender、Duration of SLE、Anti-phospholipid antibodies、Vasculitis、Maximum Daily Dose of GC >50mg、Glucocorticoid pulse therapy、Cumulative dose of GC、GC Starting Dose
Tang FL (37)	China/1999	Case control	69	29	glucocorticoids	/	X-ray	Gender、Anti-phospholipid antibodies、Hypertension、Vasculitis、Raynaud's Phenomenon、Glucocorticoid pulse therapy、GC Starting Dose
Uea-areewongsa P (38)	Thailand/2009	Case control	40	20	glucocorticoids	/	X-ray or MRI	Age at SLE Diagnosis、Gender、Duration of SLE、TG、TC、HDL-C、LDL-C、Daily GC Consumption、Maximum Daily Dose of GC >50mg、GC treatment duration、Cumulative dose of GC
Wang DX (39)	China/2009	Case control	96	32	glucocorticoids	/	X-ray, CT or MRI	Gender、Duration of SLE、Anti-phospholipid antibodies、Hypertension、Vasculitis、Raynaud's Phenomenon、Glucocorticoid pulse therapy、Initial Gc dose >60 mg/d、Cumulative dose of GC
Wang MC (40)	China/2018	Case control	86	36	glucocorticoids	/	/	Gender、BMI、Duration of SLE、TC、TG、HDL-C、LDL-C、Arthritis、Glucocorticoid pulse therapy、GC Starting Dose、SLEDAl score
Wang X (41)	China/2024	Case control	68	17	prednisone	/	X-ray or MRI	Age at SLE Diagnosis、Gender、BMI 、Duration of SLE、GC treatment duration、Glucocorticoid pulse therapy、initial Gc dose > 60 mg/d、GC Starting Dose、SLEDA score
Xuan JM (42)	China/2011	Case control	111	37	glucocorticoids	prednisone	/	Age at SLE Diagnosis、Thrombocytopenia、Arthritis、Raynaud's Phenomenon、Glucocorticoid pulse therapy、Initial Gc dose > 60 mg/d、GC Starting Dose、SLEDAl score
Xu WB (43)	China/2025	Case control	914	100	glucocorticoids	/	X-ray, CT or MRI	Age at SLE Diagnosis、Gender、Duration of SLE、Maximum Daily Dose of Gc >50mg、Glucocorticoid pulse therapy、SLEDA score
Zheng ZH (44)	China/2006	Case control	139	59	glucocorticoids	/	/	Gender、GC Starting Dose
Cheng C (45)	China/2023	Cohort	4091	2.59%	glucocorticoids	prednisone	X-ray, CT or MRI	Age at SLE Diagnosis、Gender、Arthritis、Duration of SLE、SLEDAl score
Chen S (10)	China/2021	Cohort	449	9.1%	glucocorticoids	/	MRI	Age at SLE Diagnosis、BMI、Gender、Daily GC Consumption、Maximum Daily Dose of GC>50mg、Cumulative dose of GC、SLEDAl score
Fialho SC (46)	Brazil/2007	Cohort	46	10	glucocorticoids	/	MRI	BMI、TC、TG、HDL-C、LDL-C、Cumulative dose of GC
Ghaleb RM (47)	Egypt/2011	Cohort	100	15	glucocorticoids	/	X-ray or MRI	Duration of SLE、SLEDAl score
Hisada R (48)	Japan/2019	Cohort	88	38	glucocorticoids	/	MRI	Gender、Glucocorticoid pulse therapy、SLEDA score
Kuroda T (49)	Japan/2015	Cohort	78	21	/	/	MRI	Age at SLE Diagnosis、Gender、BMI、TG、TC、HDL-C、LDL-C、Daily GC consumption、Glucocorticoid pulse therapy、SLEDAl score
Kwon H H (50)	Korea/2018	Cohort	877	113	glucocorticoids	prednisone	X-ray, CT or MRI	Age at SLE Diagnosis、Gender、Daily GC consumption、Maximum Daily Dose of GC>50mg、Glucocorticoid pulse therapy、Cumulative dose of GC、SLEDAl score
Xu YJ (51)	China/2023	Cohort	449	41	glucocorticoids	prednisone	MRI	Age at SLE Diagnosis、Gender、BMI、Daily GC consumption、Maximum Daily Dose of GC>50mg、Cumulative dose of GC、SLEDAl score

### Meta-analysis

#### Age at SLE diagnosis

**Figure 2:**
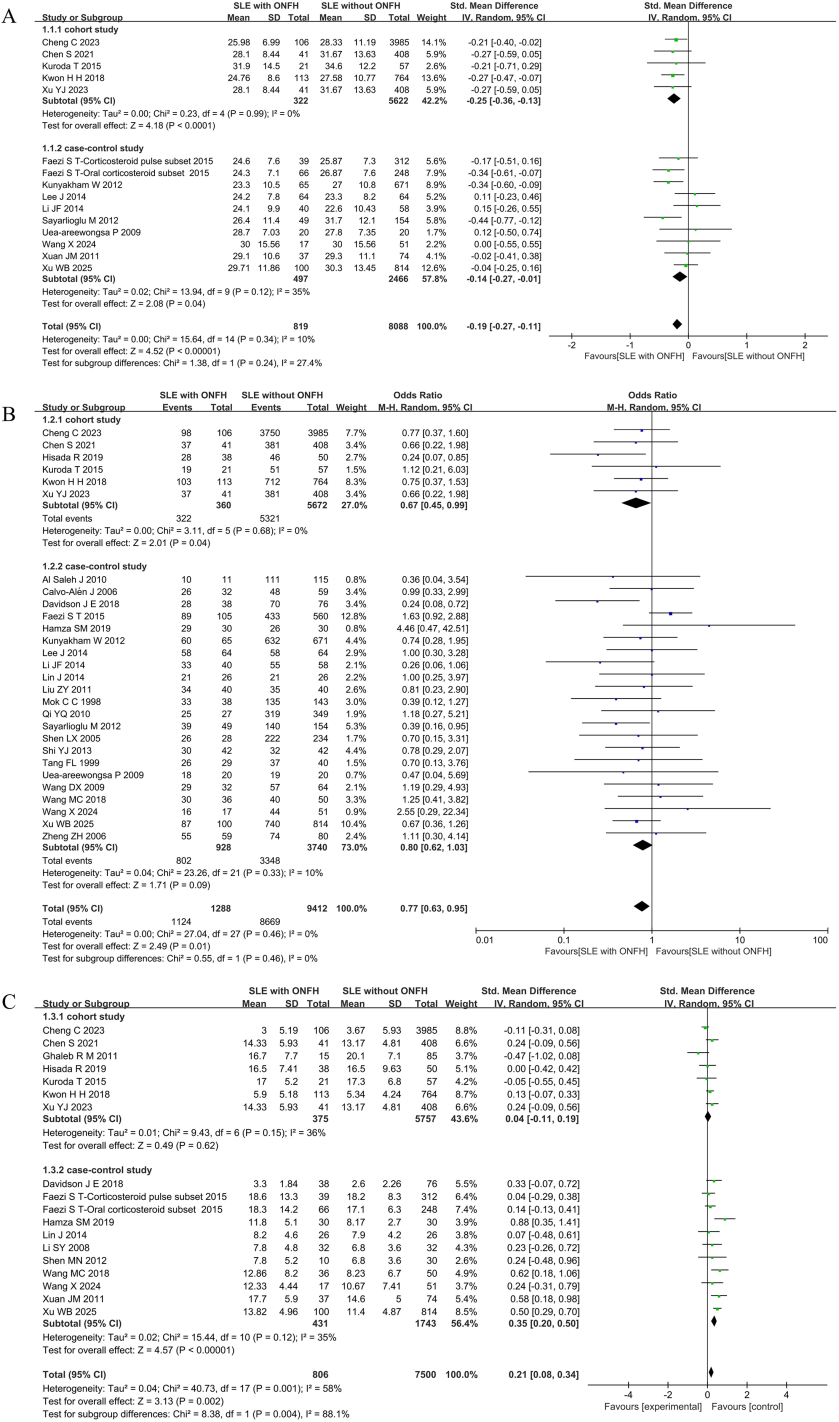
Forest plot: **(A)** Age at SLE Diagnosis; **(B)** Gender(female); **(C)** SLEDAI Score.

Fourteen of the 35 included studies (10, 21, 25–27, 33, 38, 41–43, 45, 49–51)investigated the association between age at SLE diagnosis and the development of ONFH. The meta-analysis demonstrated that patients who developed ONFH were diagnosed with SLE at a significantly younger age compared to those without ONFH, with low heterogeneity among studies [*I*^2^ = 10%, SMD = -0.19(-0.27,-0.11), *P* < 0.00001], indicating a statistically significant association. Subgroup analyses further confirmed this association, revealing a consistently younger age at SLE diagnosis in the ONFH group across both cohort and case–control studies, with all subgroup results reaching statistical significance. (**Figure 2A**)

#### Gender(female)

This study investigated the association between female gender and the development of ONFH in patients with SLE. A total of 28 studies (10, 18–21, 24–28, 30–34, 36–41, 43–45, 48–51)were included in the meta-analysis. The pooled result showed a statistically significant association, with an OR of 0.77(0.63, 0.95), *P* = 0.01, *I*^2^ = 0%. This indicates that females had 78% of the risk of developing ONFH compared to males, a finding consistent with general ONFH epidemiology. In subgroup analyses, a similar protective association was observed in cohort studies [*I*²= 0%, OR = 0.67(045, 0.99), *P* = 0.04], whereas no significant difference was found in case-control studies (**Figure 2B**).

#### SLEDAI score

This study examined the relationship between the SLEDAI Score and the occurrence of ONFH, incorporating 17 studies (10, 20, 21, 24, 28, 29, 35, 40,–43, 45, 47–51). Results showed that SLE patients with ONFH had significantly higher SLEDAI scores than those without ONFH [*I*²= 58%, SMD = 0.21(0.08, 0.34), *P* = 0.002], with statistically significant differences. However, subgroup analysis revealed no statistically significant difference in SLEDAI scores between the two groups in cohort studies [*I*²= 36%, SMD = 0.04(-0.11, 0.19), *P* = 0.62]. In case-control studies, SLE patients with concurrent ONFH had higher SLEDAI scores [*I*²=35%, SMD = 0.35(0.20, 0.50), *P* < 0.00001]. (**Figure 2C**).

#### Triglyceride (mg/dL)

**Figure 3:**
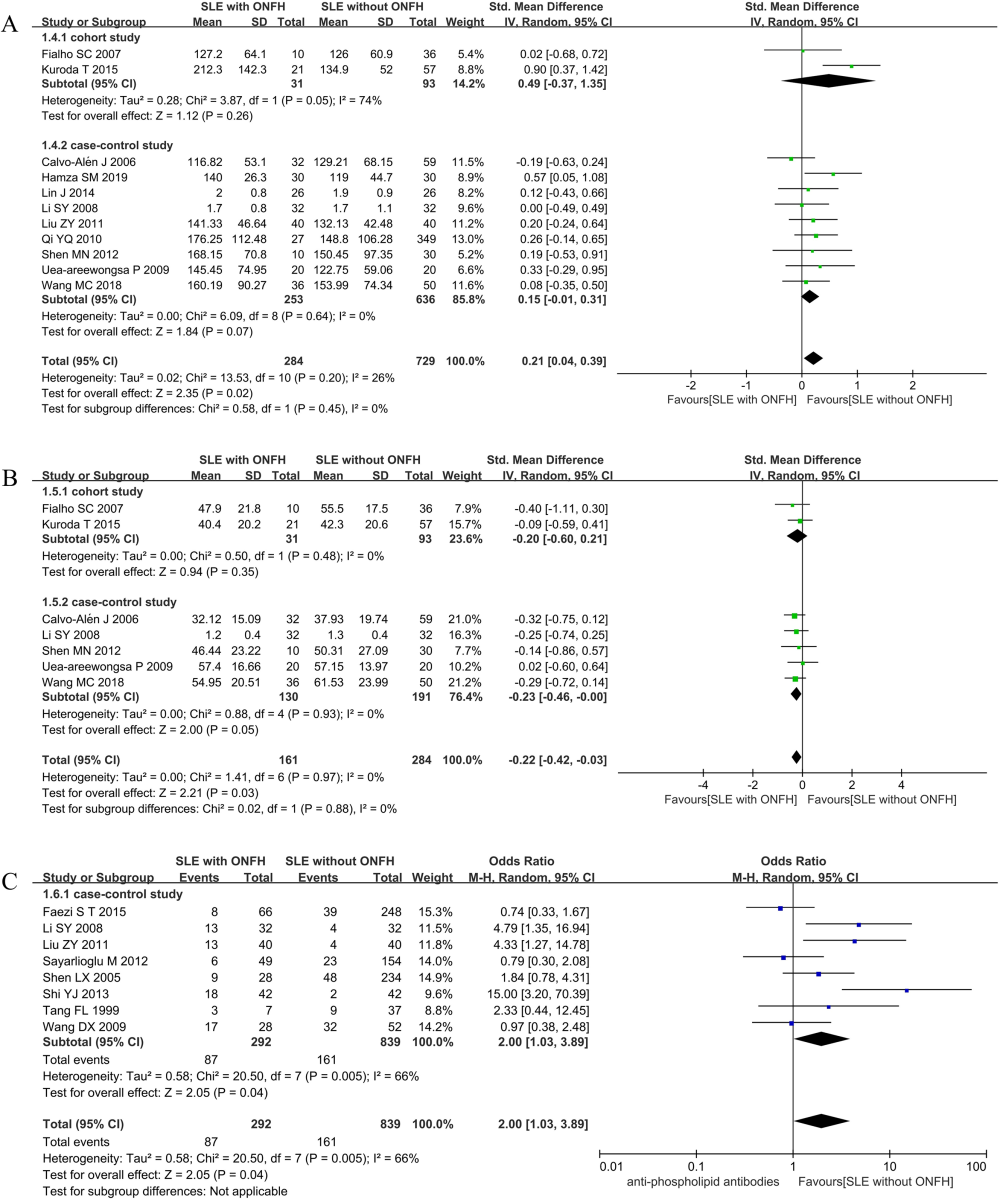
Forest plot: **(A)** Triglyceride; **(B)** HDL-C; **(C)** Anti-phospholipid antibodies.

Eleven studies (19, 24, 28–30, 32, 35, 38, 40, 46, 49) investigated the relationship between triglyceride (TG) levels and ONFH development. The pooled analysis demonstrated that SLE patients with ONFH had significantly higher TG levels than those without ONFH [*I*²= 26%, SMD = 0.21(0.04, 0.39), *P* = 0.02], with statistically significant differences. However, subgroup analyses stratified by study design found no statistically significant differences in TG levels between the two groups in either cohort or case-control studies. (**Figure 3A**).

#### High-density lipoprotein cholesterol (mg/dL)

Seven studies (19, 29, 35, 38, 40, 46, 49) examined the relationship between high-density lipoprotein cholesterol (HDL-C) levels and ONFH development. The meta-analysis revealed that SLE patients with ONFH had significantly lower HDL-C levels compared to those without ONFH [*I*²= 0%, SMD = -0.22(0.42, -0.03), *P* = 0.03]. In subgroup analyses, however, results varied: the difference in case-control studies barely reached statistical significance [*I*^2^ = 0%, SMD = -0.23(0.46, -0.00), *P* = 0.05], whereas no statistically significant difference was observed in cohort studies (**Figure 3B**).

#### Anti-phospholipid antibodies

Eight case-control studies (21, 29, 30, 33, 34, 36, 37, 39) evaluated the association between anti-phospholipid antibodies and ONFH development. The pooled result indicated that SLE patients with anti-phospholipid antibodies had a two-fold increased risk of developing ONFH [*I*²= 66%, OR = 2.00(1.03, 3.89), *P* = 0.04]. (**Figure 3C**).

#### GCs pulse therapy

**Figure 4:**
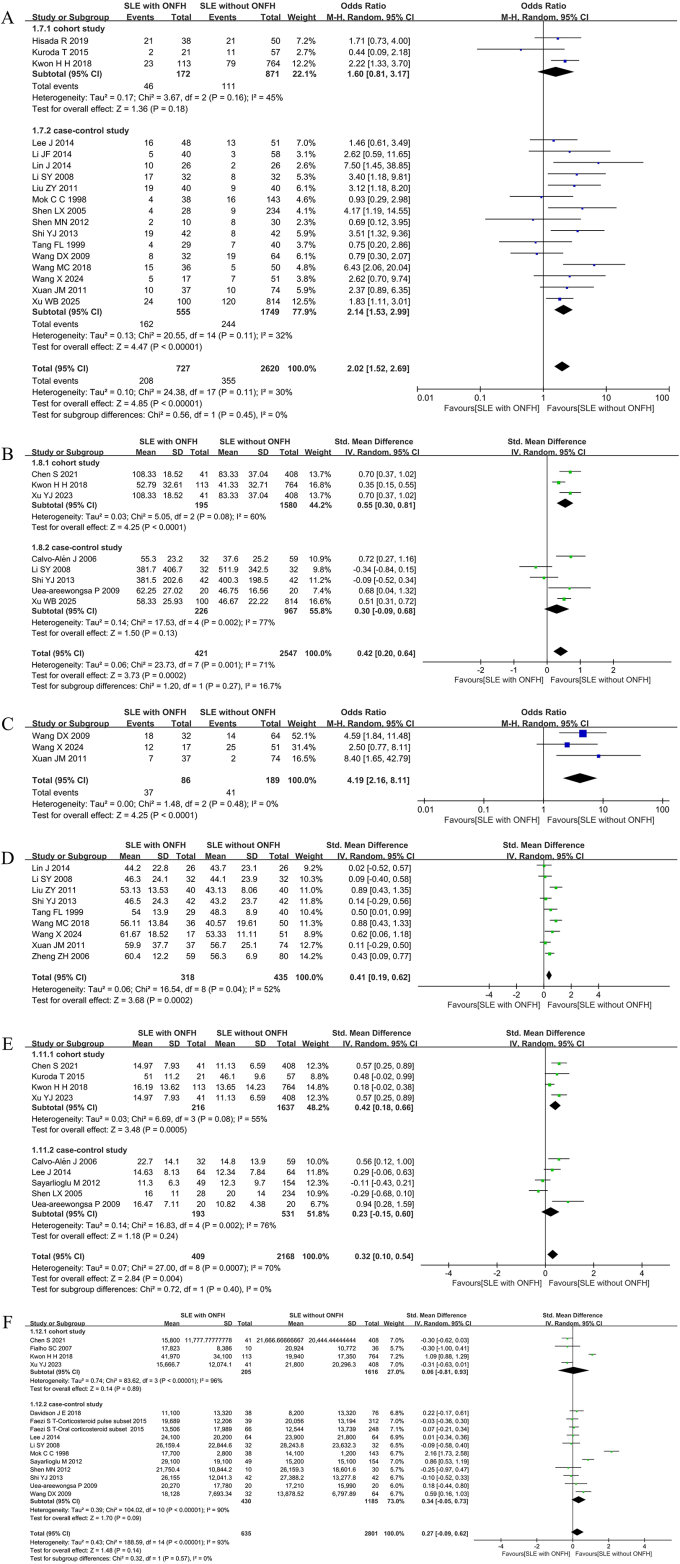
Forest plot: **(A)** GC pulse therapy; **(B)** Maximum Daily Dose of GCs(> 50 mg); **(C)** Initial GCs dose (> 60 mg/d); **(D)** GC Starting Dose(mg); **(E)** Daily GC Consumption(mg); **(F)** Cumulative dose of GCs(mg).

Eighteen studies (26–31, 34–37, 39–43, 48–50) investigated the relationship between GCs pulse therapy and ONFH risk. The overall analysis demonstrated that SLE patients receiving GCs pulse therapy had a 2.02-fold increased risk of ONFH [*I*^2^ = 30%, OR = 2.02(1.52, 2.69), *P* < 0.00001]. Subgroup analysis revealed a significant association between GCs pulse therapy and ONFH risk in case-control studies [*I*^2^ = 32%, OR = 2.14(1.53, 2.99), *P* < 0.00001], whereas no statistically significant association was observed in cohort studies. (**Figure 4A**).

#### Maximum daily dose of GCs(> 50 mg)

Eight studies (10, 19, 29, 36, 38, 43, 50, 51) evaluated the association between a maximum daily GCs dose >50 mg and ONFH development. The pooled analysis demonstrated that a maximum daily GCs dose exceeding 50 mg significantly increased the risk of ONFH in SLE patients [*I*²=71%, SMD = 0.42(0.20, 0.64), *P* = 0.0002]. In subgroup analyses, cohort studies showed a significant association [*I*^2^ = 60%, SMD = 0.55(0.30, 0.81), *P* < 0.0001], whereas no statistically significant difference was observed in case-control studies. (**Figure 4B**).

#### Initial GCs dose (> 60 mg/d)

Three case-control studies (39, 41, 42) investigated the relationship between the initial GC dose and ONFH risk in SLE patients. The meta-analysis revealed that patients with an initial GCs dose >60 mg/day had a 4.19-fold increased risk of developing ONFH [*I*²= 0%, OR = 4.19(2.16, 8.11), *P* < 0.0001]. (**Figure 4C**).

#### GCs starting dose(mg)

Data from nine case-control studies (28–30, 36, 37, 40–42, 44) examined the relationship between the GCs starting dose and ONFH development in SLE patients. The meta-analysis demonstrated that the GCs starting dose was significantly higher in SLE patients who developed ONFH compared to those who did not [*I*^2^ = 52%, SMD = 0.41(0.19, 0.62), *P* = 0.0002], indicating a statistically significant difference. (**Figure 4D**).

#### Daily GCs consumption(mg)

Nine studies (10, 19, 26, 33, 34, 38, 49–51) investigated the association between daily GCs consumption and ONFH risk. The overall analysis revealed that SLE patients who developed ONFH were administered significantly higher daily GCs doses [*I*^2^ = 70%, SMD = 0.32(0.10, 0.54), *P* = 0.004]. In subgroup analyses, cohort studies showed a significant association [*I*^2^ = 55%, SMD = 0.42(0.18, 0.66), *P* = 0.0005], whereas no statistically significant difference was observed in case-control studies [*I*² = 76%, SMD = 0.23(-0.15, 0.60), *P* = 0.24]. (**Figure 4E**).

#### Cumulative dose of GCs(mg)

A total of 14 studies (10, 20, 21, 26, 29, 31, 33, 35, 36, 38, 39, 46, 50, 51) examined the association between cumulative GCs dose and ONFH development. The pooled analysis showed no statistically significant difference in cumulative GCs dose between SLE patients with and without ONFH [*I*^2^ = 93%, SMD = 0.27(-0.09, 0.62), *P* = 0.14]. Subgroup analyses confirmed no significant association in either cohort or case-control studies. (**Figure 4F**).

#### Vasculitis

**Figure 5:**
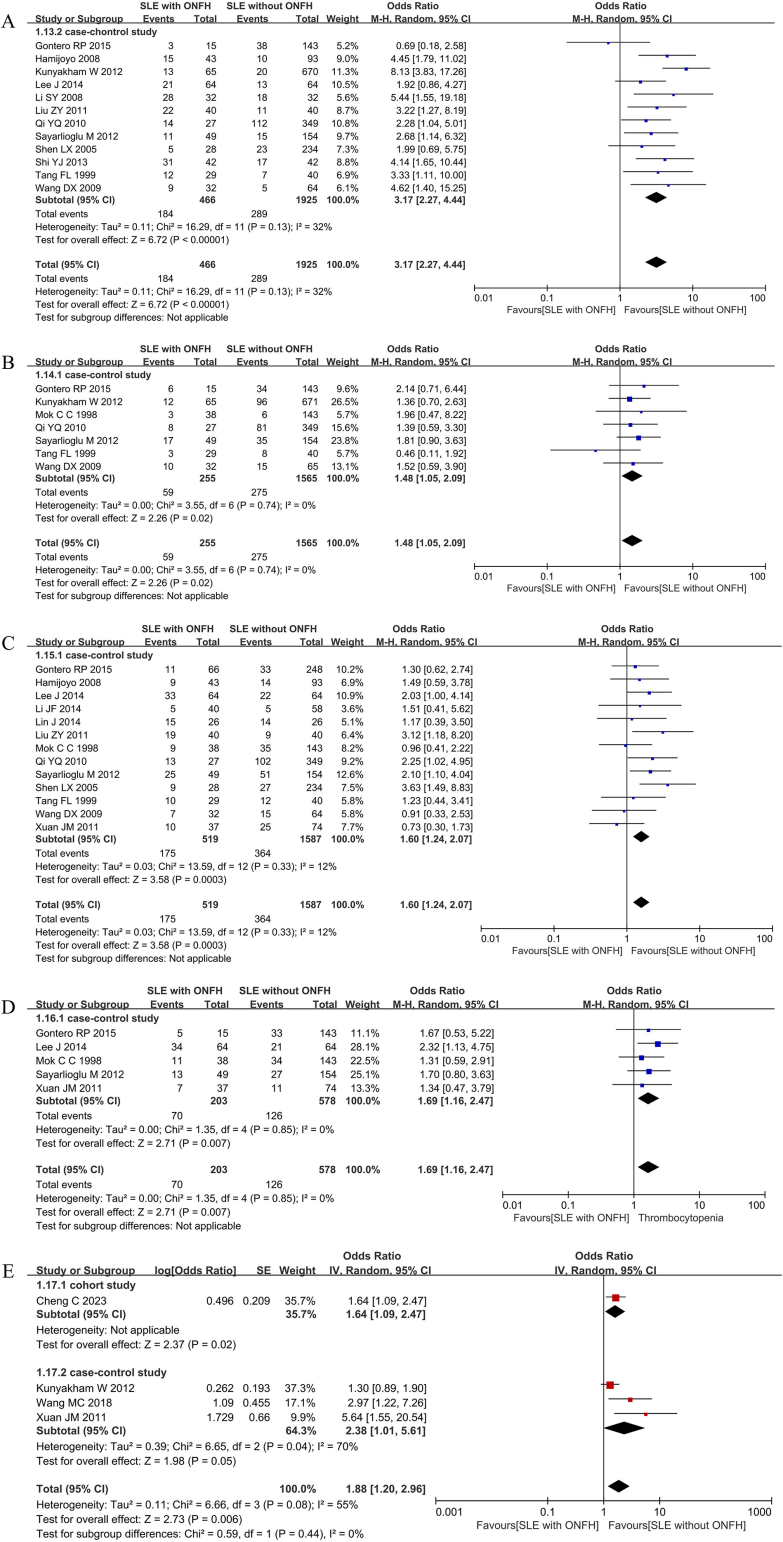
Forest plot: **(A)** Vasculitis; **(B)** Hypertension; **(C)** Raynaud’s Phenomenon; **(D)** Thrombocytopenia; **(E)** Arthritis.

Twelve case-control studies (22, 23, 25, 26, 29, 30, 32–34, 36, 37, 39) evaluated the relationship between vasculitis and ONFH risk. The meta-analysis demonstrated that SLE patients with vasculitis had a 3.17-fold increased risk of developing ONFH [*I*^2^ = 32%, OR = 3.17(2.27, 4.44), *P* < 0.00001]. (**Figure 5A**).

#### Hypertension

Data from seven case-control studies (22, 25, 31–33, 37, 39) were analyzed to assess the association between hypertension and ONFH. The results indicated that hypertensive SLE patients had a 1.48-fold higher risk of ONFH [*I*^2^ = 0%, OR = 1.48(1.05, 2.09), *P* = 0.02]. (**Figure 5B**).

#### Raynaud’s phenomenon

Thirteen case-control studies (22, 23, 26–28, 30–34, 37, 39, 42) investigated Raynaud’s phenomenon in relation to ONFH. The pooled results showed that SLE patients with Raynaud’s phenomenon had a 1.60-fold increased risk of ONFH [*I*^2^ = 12%, OR = 1.60(1.24, 2.07), *P* = 0.0003]. (**Figure 5C**).

#### Thrombocytopenia

The association between thrombocytopenia and ONFH risk was examined in five case-control studies (22, 26, 31, 33, 42).The analysis revealed that SLE patients with thrombocytopenia had a 1.69-fold increased risk of developing ONFH [*I*^2^ = 0%, OR = 1.69(1.16, 2.47), *P* = 0.007]. (**Figure 5D**).

#### Arthritis

Across four studies (25, 40, 42, 45), arthritis was identified as a significant risk factor for ONFH, with a pooled OR of 1.88 (95%CI 1.20-2.96), *I*²= 55%, *P* = 0.006. Subgroup analysis of the single included cohort study showed an OR of 1.64 (95%CI 1.09-2.47), while the three case-control studies yielded a pooled OR of 2.38 (95%CI 1.01-5.61), *I*^2^ = 70%, *P* = 0.05. (**Figure 5E**).

#### Other factors

Meta-analyses were also performed for several additional factors, including body mass index (10, 19, 40, 41, 46, 49, 51)(**Supplementary Figure S1A**), total cholesterol (19, 24, 28–30, 32, 35, 38, 40) (**Supplementary Figure S1B**), LDL-C (19, 29, 32, 35, 38, 40) (**Supplementary Figure S1C**), Duration of SLE (19, 20, 24–26, 29, 31, 32, 34, 35, 38–41, 43, 45, 47) (**Supplementary Figures S1D, E**), and GCs treatment duration (26, 31, 33, 34, 38, 41)(**Supplementary Figure S1F**). No significant associations were observed between these factors and ONFH development in SLE patients. (**Supplementary Material 3**)

### Sensitivity analysis and publication bias

Sensitivity analysis was performed using the leave-one-out method for four outcomes exhibiting substantial heterogeneity: anti-phospholipid antibodies, maximum daily GC dose (>50 mg), daily GC consumption, and cumulative GC dose. The results demonstrated that the pooled estimates remained stable for all outcomes except anti-phospholipid antibodies, where exclusion of individual studies led to meaningful changes in the effect size (**Supplementary Material 2: Table S3**). For outcomes with more than 10 included studies, funnel plots showed a relatively symmetrical distribution of effect estimates, suggesting a low risk of publication bias. (**Supplementary Material 3: Figure S2**).

## Discussion

The pathogenesis of ONFH in SLE is complex. Although the use of GCs has long been considered the primary risk factor (5), controversies persist regarding the daily dose, cumulative dose, administration route, and duration of therapy (12, 14). The inconsistency and insufficiency of such evidence hinder the development of evidence-based guidance for clinical risk stratification and medication management. Consequently, clinicians face challenges in accurately assessing the individual risk of ONFH when weighing disease control against adverse effects. This not only complicates early diagnosis and prevention but also results in some patients missing the optimal window for intervention. To address these gaps, this study synthesizes existing evidence via a systematic review and meta-analysis, aiming to identify the demographic characteristics and GC dosing patterns in SLE patients with ONFH, and to explore the association between other potential risk factors and the occurrence of ONFH.

This study integrated data from 35 studies involving 11,356 patients and systematically investigated the demographic characteristics, GC dosage, and potential risk factors for ONFH in SLE. Regarding demographic characteristics, a significant association was observed between the age at SLE diagnosis and ONFH occurrence: patients with ONFH were diagnosed with SLE at a significantly younger age than those without. This association may be attributed to two hypothetical mechanisms: first, younger individuals have more active bone metabolism, where GCs may exert a stronger effect on inducing the differentiation of bone marrow mesenchymal stem cells into adipocytes—subsequently contributing to intraosseous fat accumulation in the femoral head and exacerbating the ischemic microenvironment (7); second, younger patients may be more likely to receive high-dose GC therapy earlier after SLE diagnosis (52). These findings suggest that enhanced imaging screening for ONFH may be warranted in young SLE patients, particularly within 24 months of initiating GC therapy (13, 53). Regarding gender, although the pooled analysis indicated a lower risk of ONFH in female SLE patients (OR = 0.78), this protective effect was not consistently confirmed across subgroup analyses of cohort and case-control studies. This divergence likely reflects the distinct epidemiological profiles of these conditions: while SLE exhibits a strong female predilection (54), ONFH is approximately three times more prevalent in males in the general population (55). Consequently, gender should not serve as a solitary prognostic marker; rather, a multifactorial risk assessment approach is warranted.

Analysis of the SLEDAI score further confirmed the correlation between disease activity and the risk of ONFH. An elevated SLEDAI score reflects a more severe inflammatory response (56), and the synergistic effect of inflammation and GCs may exacerbate microcirculatory disturbances in the femoral head (57). The lack of statistical significance observed in the cohort study subgroup might stem from the rigorous monitoring inherent to prospective designs. Such protocols facilitate timely therapeutic adjustments to dampen disease flares, potentially masking the prognostic impact of high SLEDAI scores in these populations. Nevertheless, the SLEDAI score should be considered a vital indicator for ONFH screening. Given that the mean SLEDAI score across the included studies was 11.8, enhanced monitoring is recommended for patients exceeding this threshold following GC administration. We also analyzed relevant metabolic parameters. In the overall pooled analysis, patients with ONFH exhibited significantly elevated TG levels and reduced HDL-C levels. However, it is crucial to note that these associations were not consistently reproduced in the subgroup analyses. For instance, the significance of TG levels was not maintained upon stratification, and reduced HDL-C levels were not statistically significant in the cohort study subgroup. These discrepancies are likely attributable to reduced statistical power resulting from sample stratification, or potentially to residual confounding where dyslipidemia serves as a surrogate marker for high-dose GCs exposure rather than an independent causal factor. Biologically, these lipid abnormalities are hypothesized to contribute to ONFH by increasing blood viscosity and promoting microthrombosis (58–61), thereby potentially exacerbating the condition. Furthermore, this study also identified antiphospholipid antibody positivity as a significant risk factor for ONFH in SLE patients. This finding aligns with the hypothesized thrombotic mechanism mediated by antiphospholipid antibodies (48, 62), providing further evidence to support their potential role in ONFH occurrence.

A critical finding of this study is the elucidation of the dose–response relationship between distinct GC administration patterns and ONFH risk. Our analysis indicates that high-intensity exposures—specifically GC pulse therapy (OR = 2.02), a maximum daily dose >50 mg (SMD = 0.42, *P* < 0.0001), and an initial dose >60 mg/day (OR = 4.19)—were significantly associated with an elevated risk of ONFH. These findings address the debate regarding “cumulative *vs.* short-term high-dose” exposure, suggesting that high-intensity, short-term GC exposure is a more potent risk determinant than cumulative dose alone, especially when compared to low cumulative doses (<5 g) (63, 64). Furthermore, the study revealed that patients with SLE complicated by ONFH had a higher daily GC dosage, further supporting the critical role of short-term high-dose GC exposure in the development of ONFH. For instance, when the daily dose in SLE patients consistently exceeds 50 mg, the risk of ONFH increases even if the cumulative dose is not significantly elevated. Notably, no significant association was observed between cumulative GC dose and ONFH occurrence (SMD = 0.27, *P* = 0.14). This result does not contradict the Association Research Circulation Osseous (ARCO) criteria, which identify a cumulative dose >2 g within three months as a risk factor (53); rather, it underscores that dose intensity over a specific period is more critical than total lifetime accumulation. Consequently, clinical management should prioritize minimizing initial and daily doses. Pulse therapy should be administered judiciously, with prompt tapering to avoid prolonged high-dose regimens. Excessively restricting the total duration of GC therapy may be less effective for prevention than strictly controlling daily dosage intensity.

Beyond corticosteroid exposure, this study identified vasculitis, hypertension, Raynaud’s phenomenon, thrombocytopenia, and arthritis as independent risk factors for ONFH in SLE. The relationship between vasculitis and ONFH likely involves a synergistic mechanism: high disease activity necessitates high-dose GC therapy, which, combined with vascular pathology, may significantly elevate ONFH risk (65). However, interpreting this result requires caution due to the inconsistent reporting of vasculitis diagnostic criteria across the included studies. Regarding hypertension, the present results indicate a significant association with ONFH, distinguishing this study from some previous reports and warranting further investigation. Raynaud’s phenomenon is caused by neuro-humoral dysregulation (66), a condition characterized by vasospasm that is hypothesized to extend beyond the extremities to the microvascular branches of the femoral head, potentially causing recurrent transient ischemia. Similarly, despite thrombocytopenia, platelets in SLE often remain activated and release procoagulant granules, which may sustain a hypercoagulable state (67, 68). Finally, arthritis-related synovial effusion (69) could theoretically increase intra-articular pressure, indirectly compromising femoral head perfusion. Given these multifactorial risks, integrating these comorbidities into clinical risk stratification models is crucial for identifying high-risk patients and implementing early interventions.

Despite the robust evidence synthesized in this meta-analysis, several limitations warrant consideration. First, moderate to high heterogeneity (*I*² = 32%-93%) was observed for certain indicators (e.g., TG, daily GC dose, cumulative GC dose). This variability is likely attributable to differences in GC standardization methods (e.g., conversion to prednisone equivalents), inconsistent diagnostic criteria for ONFH (e.g., MRI *vs.* X-ray), and racial diversity among the included populations. Second, selection bias is a potential concern in the retrospective case-control studies, as control groups may not have been rigorously matched for key confounders such as disease activity and prior GC exposure. Third, for outcomes where overall significance was not maintained in subgroup analyses, the possibility of limited statistical power within subgroups and residual confounding cannot be excluded. Finally, potential synergistic interactions among risk factors—such as the combined impact of male sex and high initial GC dosage—could not be evaluated due to the lack of individual patient data. Given these constraints, future research should prioritize multicenter prospective cohort studies with rigorous control of confounding variables to clarify the independent effects and interactions of risk factors. Concurrently, steroid-sparing regimens and preventive strategies should be explored for high-risk patients to minimize ONFH occurrence.

## Conclusion

This study identified the key high-risk population characteristics, core management directions for GC therapy, and priorities for risk prevention and control of SLE-associated ONFH. These characteristics include age, SLEDAI score, abnormal levels of TG and HDL-C, and positivity for antiphospholipid antibodies. Concurrently, the presence of complications such as vasculitis, hypertension, Raynaud’s phenomenon, thrombocytopenia, or arthritis can serve as characteristic indicators for screening high-risk ONFH populations. Furthermore, it is recommended that after SLE diagnosis and initiation of GC therapy, continuous imaging screening for ONFH be performed. Particularly for patients receiving high initial GC doses or pulse therapy, the screening frequency should be appropriately increased to facilitate early detection and intervention. Regarding GC administration strategies, the principle of using the lowest effective dose should be adhered to, avoiding unnecessary short-term exposure to high-dose GCs. This study provides practical guidance for the clinical risk management of ONFH in SLE patients; however, management strategies still require dynamic adjustment based on individual patient conditions. Future multicenter prospective studies may further validate the interactions among various risk factors, optimize the risk scoring system, and promote the development of more precise and individualized prevention and control strategies.

## Data Availability

The raw data supporting the conclusions of this article will be made available by the authors, without undue reservation.
