# Supplementary figures and images for: Population characteristics, glucocorticoid dosage, and risk factors for osteonecrosis of the femoral head in systemic lupus erythematosus: a Systematic Review and meta-analysis
Source: Front Immunol. 2026 Feb 11;17:1755818. doi: 10.3389/fimmu.2026.1755818 (PMC12932495; doi:10.3389/fimmu.2026.1755818)

Figure S1

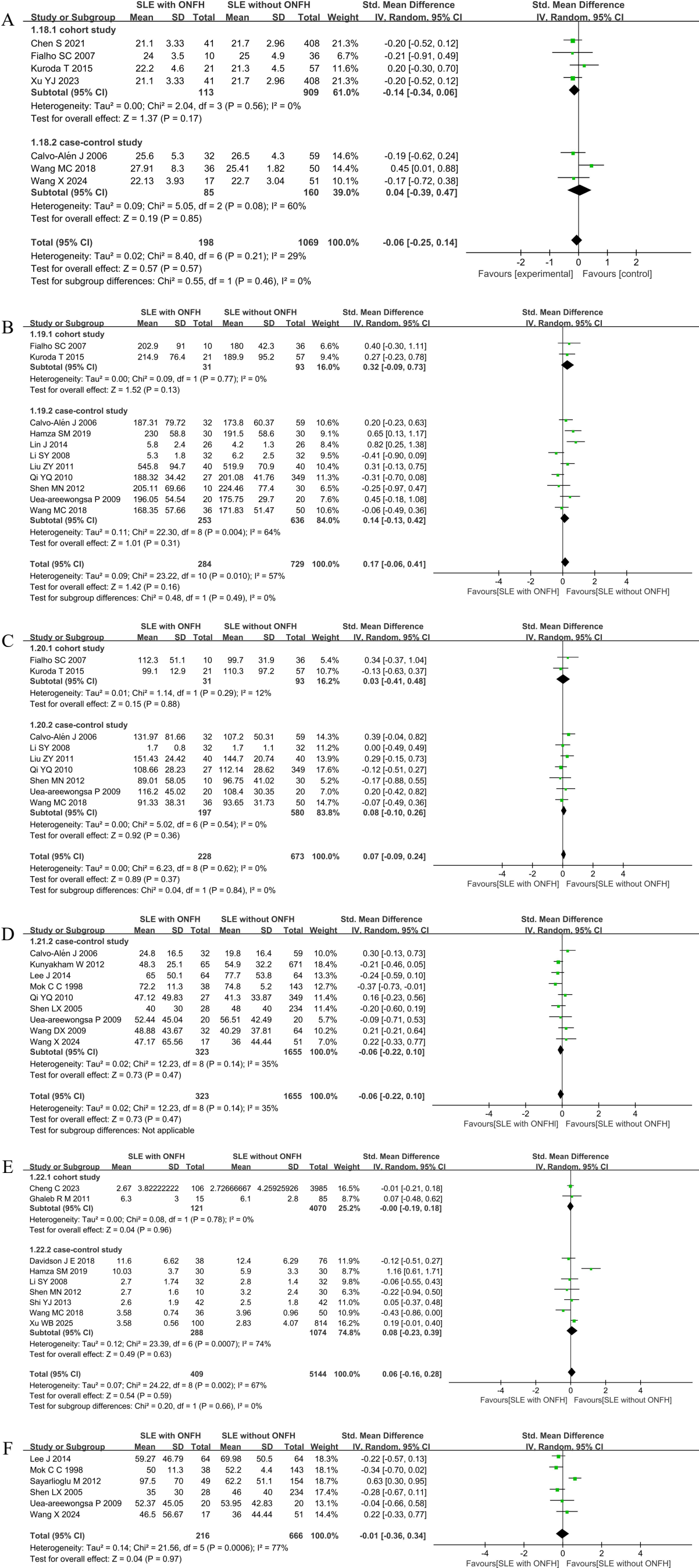

Figure S2

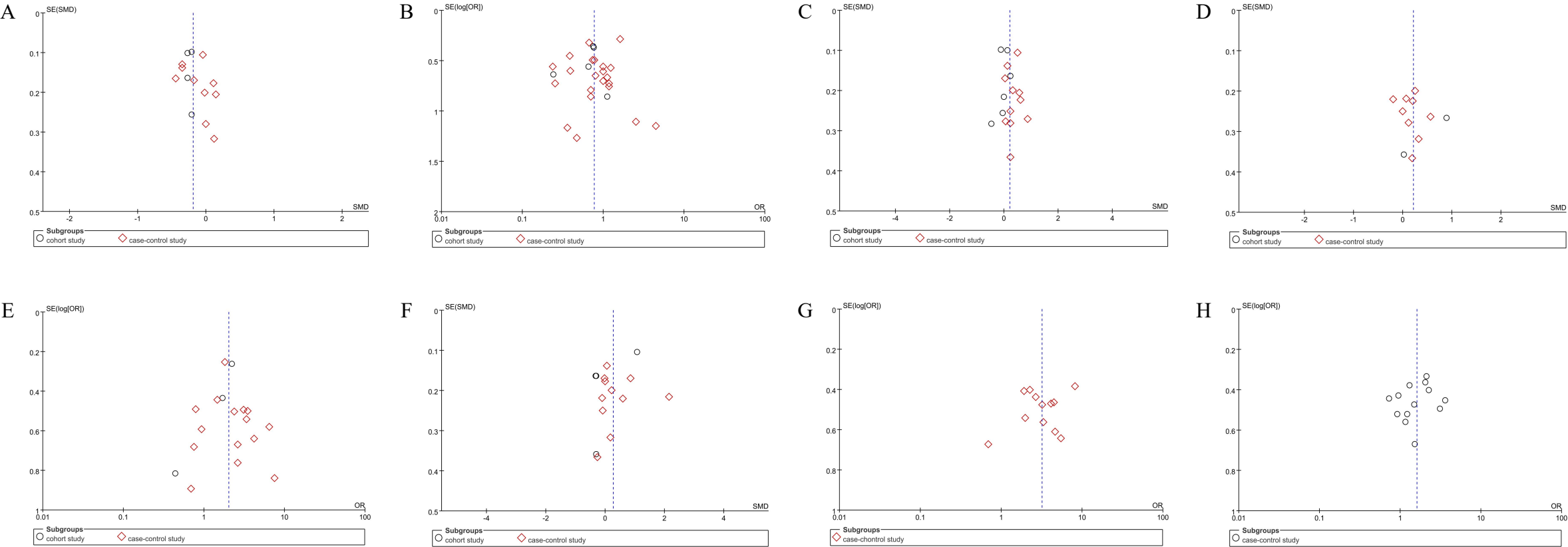

Supplement: Supplementary file 1 [file DataSheet1.pdf]
