# Supplementary material for: Population characteristics, glucocorticoid dosage, and risk factors for osteonecrosis of the femoral head in systemic lupus erythematosus: a Systematic Review and meta-analysis
Source: Front Immunol. 2026 Feb 11;17:1755818. doi: 10.3389/fimmu.2026.1755818 (PMC12932495; doi:10.3389/fimmu.2026.1755818)
Supplement: Supplementary file 2 [file DataSheet2.docx]

**Table S1 Quality Assessment of 27 Case-Control Studies**

| Study | Selection | | | | Comparability | Exposure | | | Scores |
| --- | --- | --- | --- | --- | --- | --- | --- | --- | --- |
|  | Is the case definition adequate? | Representativeness of the cases | Selection of Controls | Definition of Controls | Comparability of cases and controls on the basis of the design or analysis | Ascertainment of exposure | Same method of ascertainment for cases and controls | Non-Response rate |  |
| Al Saleh J 2010 | ★ | ★ | ☆ | ★ | ☆☆ | ☆ | ★ | ☆ | 4 |
| Calvo-Alén J 2006 | ★ | ★ | ★ | ★ | ★★ | ★ | ★ | ★ | 9 |
| Davidson J E 2018 | ★ | ★ | ☆ | ★ | ★★ | ★ | ★ | ★ | 8 |
| Faezi S T 2015 | ★ | ★ | ☆ | ★ | ★ | ★ | ★ | ☆ | 6 |
| Gontero RP 2015 | ★ | ★ | ☆ | ★ | ★★ | ★ | ★ | ☆ | 7 |
| HAMIJOYO L 2008 | ★ | ★ | ☆ | ★ | ★ | ★ | ★ | ☆ | 6 |
| Kunyakham W 2012 | ★ | ★ | ☆ | ★ | ★ | ☆ | ★ | ☆ | 5 |
| Hamza SM 2019 | ★ | ★ | ☆ | ★ | ☆☆ | ☆ | ★ | ☆ | 4 |
| Lee J 2014 | ★ | ★ | ☆ | ★ | ☆☆ | ★ | ★ | ☆ | 5 |
| Li JF 2014 | ★ | ★ | ☆ | ★ | ★ | ★ | ★ | ☆ | 6 |
| Lin J 2014 | ★ | ☆ | ☆ | ★ | ☆☆ | ☆ | ★ | ☆ | 3 |
| Li SY 2008 | ★ | ★ | ☆ | ★ | ☆☆ | ☆ | ★ | ☆ | 4 |
| Liu ZY 2011 | ★ | ★ | ☆ | ★ | ★ | ★ | ★ | ☆ | 6 |
| Mok C C 1998 | ★ | ★ | ☆ | ★ | ☆☆ | ☆ | ★ | ☆ | 3 |
| Qi YQ 2010 | ★ | ★ | ☆ | ★ | ★ | ★ | ★ | ☆ | 6 |
| Sayarlioglu M 2012 | ★ | ★ | ☆ | ★ | ☆☆ | ★ | ★ | ☆ | 5 |
| Shen LX 2005 | ★ | ☆ | ☆ | ★ | ☆☆ | ☆ | ★ | ☆ | 3 |
| Shen MN 2012 | ★ | ★ | ☆ | ★ | ★ | ☆ | ★ | ☆ | 5 |
| Shi YJ 2013 | ★ | ★ | ☆ | ★ | ☆☆ | ☆ | ★ | ☆ | 4 |
| Tang FL 1999 | ★ | ★ | ☆ | ★ | ★ | ☆ | ★ | ☆ | 5 |
| Uea-areewongsa P 2009 | ★ | ★ | ☆ | ★ | ★★ | ★ | ★ | ☆ | 7 |
| Wang DX 2009 | ★ | ★ | ☆ | ★ | ★ | ★ | ★ | ☆ | 6 |
| Wang MC 2018 | ★ | ★ | ☆ | ★ | ★ | ★ | ★ | ☆ | 6 |
| Wang X 2024 | ★ | ★ | ☆ | ★ | ☆☆ | ★ | ★ | ☆ | 5 |
| Xuan JM2011 | ★ | ★ | ☆ | ★ | ☆☆ | ☆ | ★ | ☆ | 5 |
| Xu WB 2025 | ★ | ★ | ☆ | ★ | ☆☆ | ★ | ★ | ☆ | 5 |
| Zheng ZH 2006 | ★ | ☆ | ☆ | ★ | ☆☆ | ☆ | ★ | ☆ | 3 |

**Table S2 Quality Assessment of 8 Cohort Studies**

| Study | Selection |  |  |  | Comparability | Exposure |  |  | Scores |
| --- | --- | --- | --- | --- | --- | --- | --- | --- | --- |
|  | Representativeness of the exposed cohort | Selection of the non exposed cohort | Ascertainment of exposure | Demonstration that outcome of interest was not present at start of study | Comparability of cohorts on the basis of the design or analysis | Assessment of outcome | Was follow-up long enough for outcomes to occur | Adequacy of follow up of cohorts |  |
| Cheng C 2023 | ★ | ★ | ★ | ★ | ☆☆ | ★ | ★ | ★ | 7 |
| Chen S 2021 | ★ | ★ | ★ | ★ | ★ | ★ | ★ | ★ | 8 |
| Fialho S 2007 | ★ | ★ | ★ | ★ | ★ | ☆ | ★ | ☆ | 6 |
| Ghaleb RM 2011 | ★ | ★ | ☆ | ★ | ☆☆ | ☆ | ★ | ☆ | 4 |
| Hisada R 2019 | ★ | ★ | ★ | ★ | ☆☆ | ☆ | ★ | ★ | 6 |
| Kuroda T 2015 | ★ | ★ | ☆ | ★ | ☆☆ | ☆ | ★ | ★ | 5 |
| Kwon H H 2018 | ★ | ★ | ★ | ★ | ☆☆ | ☆ | ★ | ☆ | 5 |
| Xu YJ 2023 | ★ | ★ | ☆ | ★ | ★ | ★ | ★ | ★ | 7 |

**Table S3 Sensitivity Analysis**

| Indicators | Preliminary results | | | Exclude the study with the highest weighting | | | Exclude studies with the lowest weighting | | |
| --- | --- | --- | --- | --- | --- | --- | --- | --- | --- |
|  | *SMD/OR* | 95%*CI* | *P* | *SMD/OR* | 95%*CI* | *P* | *SMD/OR* | 95%*CI* | *P* |
| Anti-phospholipid antibodies | 2.00 | 1.03-3.89 | 0.04 | 2.39 | 1.17-4.86 | 0.02 | 1.99 | 0.96-4.12 | 0.06 |
| Maximum Daily Dose of GC(> 50 mg) | 0.42 | 0.20-0.64 | 0.0002 | 0.43 | 0.15-0.70 | 0.002 | 0.40 | 0.16-0.63 | 0.0009 |
| Daily GC Consumption | 0.32 | 0.10-0.54 | 0.004 | 0.35 | 0.08-0.61 | 0.01 | 0.27 | 0.06-0.49 | 0.01 |
| Cumulative dose of GC | 0.27 | -0.09-0.62 | 0.14 | 0.20 | -0.13-0.54 | 0.24 | 0.30 | -0.07-0.66 | 0.11 |
